# Stroke Admissions, Stroke Severity, and Treatment Rates in Urban and Rural Areas During the COVID-19 Pandemic

**DOI:** 10.3389/fneur.2020.607193

**Published:** 2021-01-06

**Authors:** Hebun Erdur, Bob Siegerink, Christoph Leithner, Christiana Franke, Irina Lorenz-Meyer, Sarah Theen, Anselm Angermaier, Stephan Kinze, Joachim E. Weber, Jessica L. Rohmann, Jan F. Scheitz, Christian H. Nolte, Matthias Endres, Heinrich J. Audebert

**Affiliations:** ^1^Klinik und Hochschulambulanz für Neurologie, Charité - Universitätsmedizin Berlin, Berlin, Germany; ^2^Center for Stroke Research Berlin, Charité – Universitätsmedizin Berlin, Berlin, Germany; ^3^Department of Neurology, University Medicine Greifswald, Greifswald, Germany; ^4^Klinik für Neurologie, Unfallkrankenhaus Berlin, Berlin, Germany; ^5^Berlin Institute of Health (BIH), Berlin, Germany; ^6^Institute of Public Health, Charité – Universitätsmedizin Berlin, Berlin, Germany; ^7^DZHK (German Center for Cardiovascular Research), partner site Berlin, Berlin, Germany; ^8^DZNE (German Center for Neurodegenerative Diseases), partner site Berlin, Berlin, Germany

**Keywords:** stroke, COVID19, SARS-CoV-2, epidemiology, public health

## Abstract

**Background:** Many regions worldwide reported a decline of stroke admissions during the early phase of the coronavirus disease 2019 (COVID-19) pandemic. It remains unclear whether urban and rural regions experienced similar declines and whether deviations from historical admission numbers were more pronounced among specific age, stroke severity or treatment groups.

**Methods:** We used registry datasets from (a) nine acute stroke hospitals in Berlin, and (b) nine hospitals from a rural TeleNeurology network in Northeastern Germany for primary analysis of 3-week-rolling average of stroke/TIA admissions before and during the COVID-19 pandemic. We compared course of stroke admission numbers with regional cumulative severe acute respiratory syndrome coronavirus 2 (Sars-CoV-2) infections. In secondary analyses, we used emergency department logs of the Berlin Charité University hospital to investigate changes in age, stroke severity, and thrombolysis/thrombectomy frequencies during the early regional Sars-CoV-2 spread (March and April 2020) and compared them with preceding years.

**Results:** Compared to past years, stroke admissions decreased by 20% in urban and 20-25% in rural hospitals. Deviations from historical averages were observable starting in early March and peaked when numbers of regional Sars-CoV-2 infections were still low. At the same time, average admission stroke severity and proportions of moderate/severe strokes (NIHSS >5) were 20 and 20–40% higher, respectively. There were no relevant deviations observed in proportions of younger patients (<65 years), proportions of patients with thrombolysis, or number of thrombectomy procedures. Stroke admissions at Charité subsequently rebounded and reached near-normal levels after 4 weeks when the number of new Sars-CoV-2 infections started to decrease.

**Conclusions:** During the early pandemic, deviations of stroke-related admissions from historical averages were observed in both urban and rural regions of Northeastern Germany and appear to have been mainly driven by avoidance of admissions of mildly affected stroke patients.

## Introduction

Authorities and governments worldwide have released public health recommendations and restrictions in order to contain the outbreak of Sars-CoV-2. Avoidance of physical contact whenever possible has been one of the most important recommendations in the early phase of the COVID-19 pandemic in Europe. Large regional outbreaks overwhelming local health systems and increasing nosocomial Sars-CoV-2 infections have alarmed the public, prompting the German government among others to issue a strict physical distancing decree on March 22, 2020 (1). Parallel to implementing lockdown rules, hospitals reported decreased admissions of patients with acute cardio- and cerebrovascular diseases in emergency departments (2). As a reaction, health providers advised the public not to avoid necessary medical attendance for chest pain or neurological deficits but patients may have chosen to stay at home because of fear of infection with Sars-CoV-2 (3).

In this descriptive study, we aimed to quantify acute stroke hospitalizations in metropolitan and rural hospitals in Northeastern Germany before and during the early local phase of the COVID-19 pandemic. We hypothesized that fewer patients with stroke and TIA presented to hospitals and that this decrease was predominantly driven by patients with mild stroke severity. In addition to stroke severity, we investigated age groups, proportions of patients who received thrombolysis, and numbers of thrombectomies.

## Methods

### Data Availability Statement

Data and analysis code will be made available upon reasonable request to the corresponding author.

### Study Design and Population

In our primary analysis, we investigated hospital admissions for ischemic stroke, hemorrhagic stroke, and TIA in both an urban and a rural setting in Berlin and Northeastern Germany, respectively. For this, we used data obtained from two data sources: (a) from nine Berlin hospitals with stroke units participating in a metropolitan stroke registry (B-SPATIAL, ClinicalTrials.gov Identifier: NCT03027453) and (b), from inpatient case data of nine hospitals participating in an acute TeleNeurology network in the rural areas of the states of Brandenburg and Mecklenburg-Western Pomerania in Northeastern Germany (ANNOTeM, German Clinical Trials Register Identifier: DRKS00013067). A complete list of participating hospitals and a map of both registries is presented in the Supplementary Material. For this study, we included all patients with a main ICD-10 diagnosis code of hemorrhagic or ischemic stroke (I60–I63) or transient ischemic attack (TIA, G45) without restriction in onset to admission times from January 1, 2018 to March 31, 2020 on an individual patient level.

For our secondary analyses, we obtained data from the emergency department logs of our tertiary care center Charité – Universitätsmedizin Berlin (Campus Benjamin Franklin and Campus Virchow Klinikum). These logs include information of all patients who were diagnosed with hemorrhagic or ischemic stroke or TIA (I60–63 and G45) by a neurologist in the emergency department. These data include routinely recorded information on age, stroke severity as assessed by National Institutes of Health Stroke Scale (NIHSS), intravenous thrombolysis, and thrombectomy. Because the weekly numbers of patients with thrombectomy were small, we provide these numbers on a per month basis. We included all stroke-related events documented in the emergency department logs from January 1, 2016 to May 13, 2020 for the current analysis.

### Statistical Analyses

Stroke data were plotted for the hospital stroke admission analyses from Jan 1, 2018 to March 31, 2020 and for the Charité emergency department stroke logs from Jan 1, 2016 to and including May 12, 2020. Calendar dates were transformed to number of days from January 1st of each year, with full weeks (1–52) defined as 7-day stretches, to facilitate comparability between years. Only full weeks were included. In the primary analysis, the data from prior years (before January 1, 2020) was used to obtain a historical reference value, which was computed as the weekly average number of admissions. In secondary analyses, we depict historical weekly averages and deviations in proportions of stroke patients <65 years, NIHSS on admission, proportion of more severe stroke (NIHSS >5), and number and proportion of patients who received intravenous thrombolysis and/or thrombectomy. Setting the weekly averages over the past years to 100%, we plotted the relative deviations for each week for 2020 as well as past years. We show the data with a rolling average with a 3 week window to reduce the noise in the data; all graphs with weekly number accompanied with the corresponding confidence interval that quantify the precision of the data are shown in the Supplementary Material.

Weekly numbers of officially confirmed Sars-CoV-2 infections and registered COVID-19 deaths in the city of Berlin and Northeastern Germany (obtained from https://www.berlin.de/corona/fallstatistik/ and https://www.rki.de/DE/Content/InfAZ/N/Neuartiges_Coronavirus/Fallzahlen.html) were overlaid on these plots for contextualization of the unfolding COVID-19 pandemic in our setting. All descriptive analyses and plots were performed in STATA 14.

### Ethics

ANNOTeM was approved by the Charité Ethics Committee (ANNOTeM: EA4/188/17). The B-SPATIAL and Charité emergency department stroke data contain prospectively collected data records for the purpose of quality improvement measures as regulated by German Social Code, Book V, §135a. The Berlin legislation for hospitals (§25 Berliner Krankenhausgesetz) allows the use of B-SPATIAL and Charité routine care and quality monitoring data for scientific evaluations and is exempt from approval by a local review board. Before using the data from B-SPATIAL, ANNOTeM, and Charité, all individual datasets were anonymized.

## Results

### Metropolitan Area of Berlin

**Figure 1:**
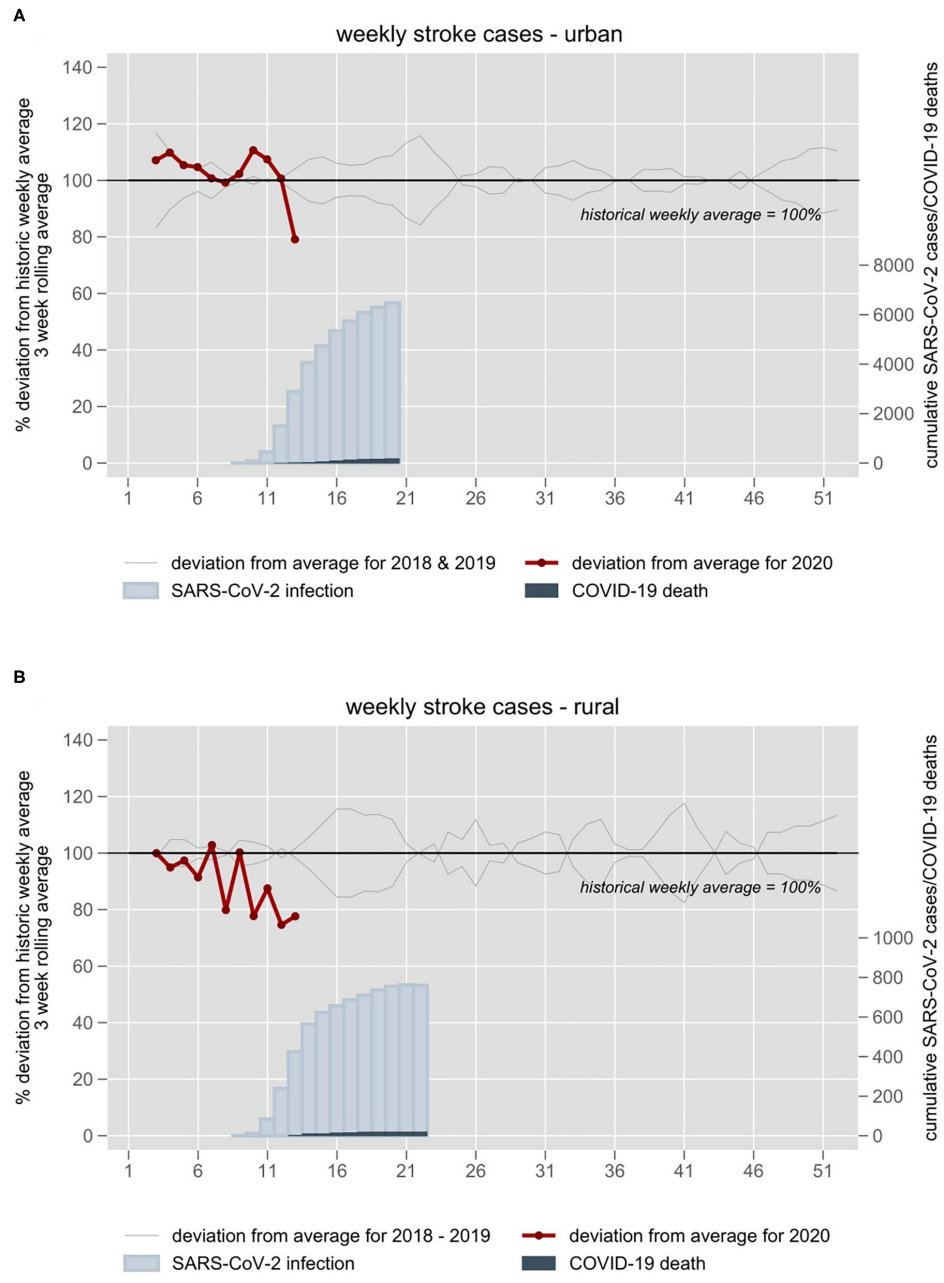
Stroke and TIA admission numbers of **(A)** nine stroke units in the metropolitan area of Berlin and **(B)** nine hospitals of a TeleNeurology Network in Northeastern Germany 2018–2020. Admission numbers are plotted against week numbers. All individual years are plotted in gray. The 3-week rolling average of 2018–2019 is set at 100%. The year 2020 is plotted in red. Blue inlays depict cumulative confirmed Sars-CoV-2 infections and COVID-19 deaths in the respective weeks.

From January 1, 2018 to March 31, 2020, a total of 19,435 patients were diagnosed and recorded with ischemic stroke, hemorrhagic stroke, or TIA in nine Berlin acute stroke hospitals participating in the B-SPATIAL registry with a weekly average of 172 (SD ±21) patients. Figure 1A shows the 3-week rolling averages of stroke and TIA cases in 2020 with a sharp deviation of 20% fewer patients compared to the past years' average recorded in week 13 (covering the period from March 18 to April 7, 2020).

### Rural Area of Northeastern Germany

A total of 3,855 patients diagnosed with ischemic stroke, hemorrhagic stroke, or TIA were admitted to one of nine ANNOTeM hospitals in Northeastern Germany from January 1, 2018 to March 31, 2020 with a weekly mean of 33 (SD ±4) cases. Figure 1B shows a negative deviation of stroke and TIA cases 20–25% compared to the weekly historical average during weeks 12 and 13.

### Charité Hospital

**Figure 2:**
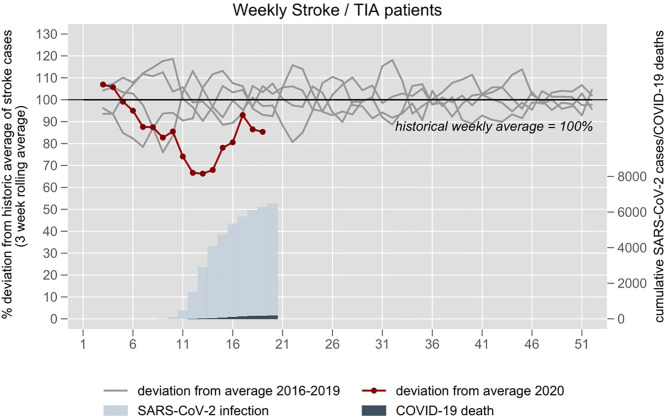
Stroke and TIA related emergency department visits at the University hospital Charité in Berlin 2016–2020. The 3-week rolling average of 2016–2019 is set at 100%. The year 2020 is plotted in red. Blue inlay depicts cumulative confirmed Sars-CoV-2 infections and COVID-19 deaths in the respective weeks.

From January 1, 2016 to May 12, 2020 two emergency departments of the Charité logged 8,779 ischemic stroke, hemorrhagic stroke, and TIA patients with a weekly mean number of 39 (SD ±6). The number of cases was 25–35% lower compared to the historical average from weeks 11 to 14 (Figure 2). The number of stroke patients increased again since week 15 but remained slightly below the historical average.

**Figure 3:**
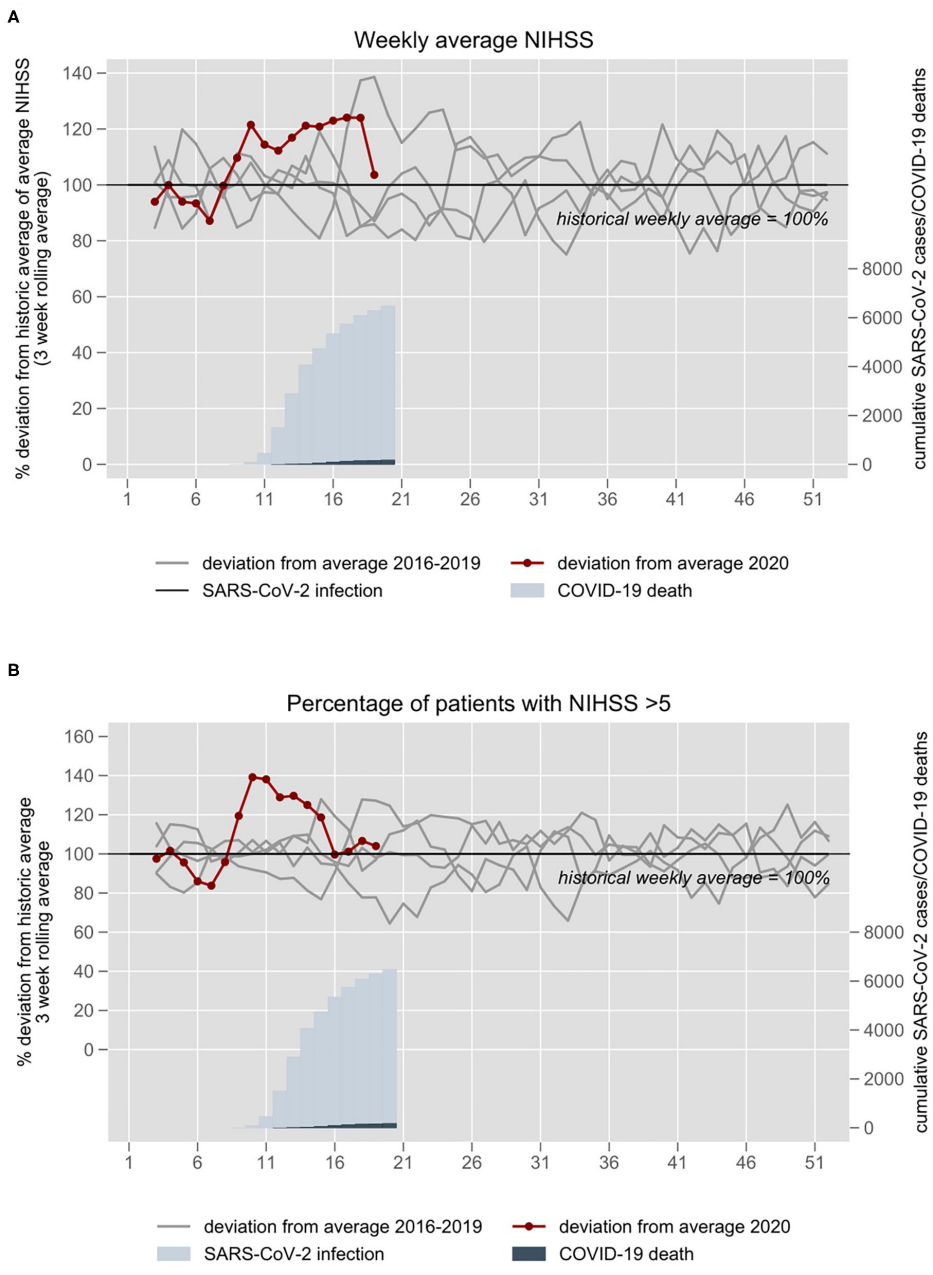
**(A)** NIHSS on admission and **(B)** proportion of patients with moderate to severe stroke (NIHSS >5) at the University hospital Charité in Berlin 2016–2020. The 3-week rolling average of 2016–2019 is set at 100%. The year 2020 is plotted in red. Blue inlays depict cumulative confirmed Sars-CoV-2 infections and COVID-19 deaths in the respective weeks.

The average of the weekly mean NIHSS on admission had been 5.5 (SD ±1.2) in the pre-pandemic period. During the pandemic period, the mean NIHSS of stroke patients was about 20% higher compared to the historical average throughout weeks 10–18 (Figure 3A). In 2020, weekly mean NIHSS in weeks 1–8 was 5.5 (SD ±0.8), in weeks 9–12 6.4 (SD ±0.6), in weeks 12–16 6.8 (SD ±1.1), and in weeks 17–19 5.8 (SD 1.2). Similarly, the proportion of patients with moderate to severe stroke (NIHSS >5) was about 20-40% higher compared to the historical average from weeks 9 to 15 but returned to the average thereafter (Figure 3B).

**Figure 4:**
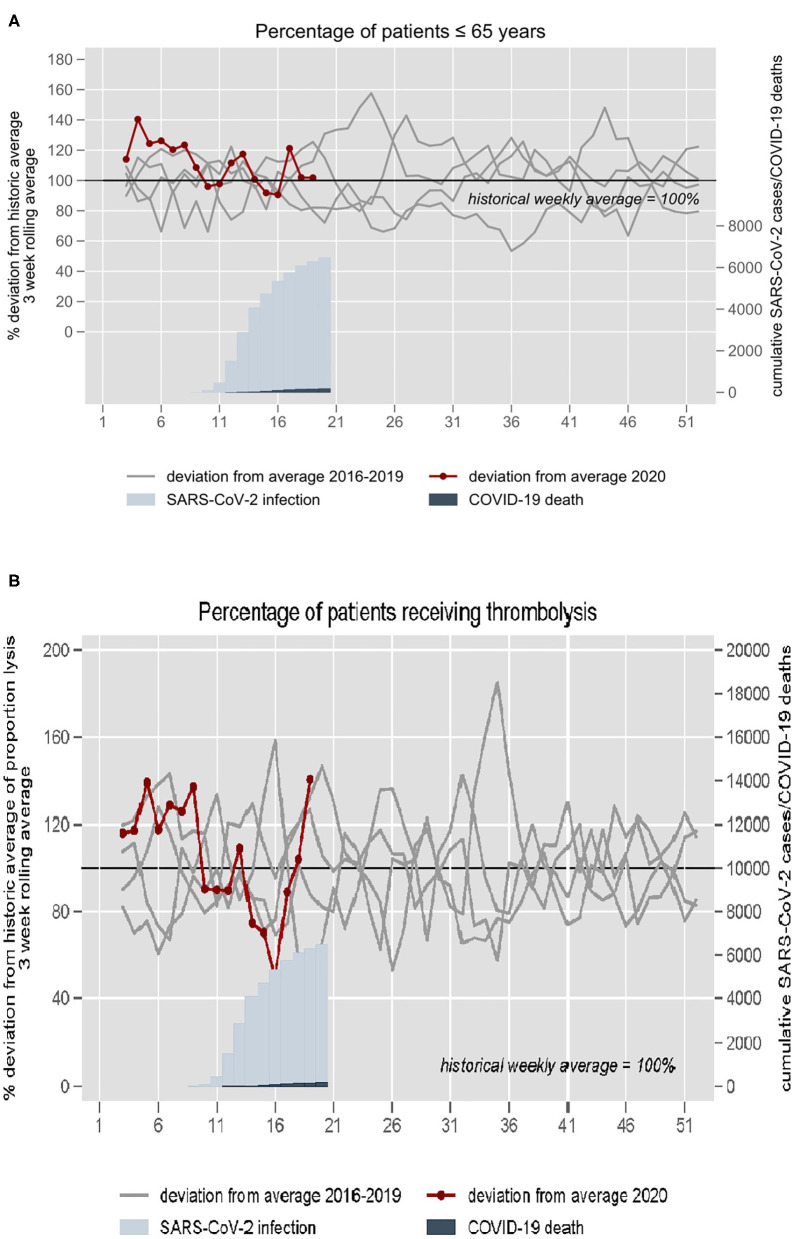
**(A)** Proportion of patients ≤65 years and **(B)** proportion of patients treated with thrombolysis at the University hospital Charité in Berlin 2016–2020. The 3-week rolling average of 2016–2019 is set at 100%. The year 2020 is plotted in red. Blue inlays depict cumulative confirmed Sars-CoV-2 infections and COVID-19 deaths in the respective weeks.

The proportion of younger patients (<65 years) was similar to the historical average (Figure 4A). In 2020, weekly mean number of patients who received thrombolysis in weeks 1–8 was 7.5 (SD ±1.7), in weeks 9–12 3.0 (SD ±1.9), and in weeks 12–16 6.7 (SD ±2.0). The proportion of patients who received thrombolysis was similar to the historical average (Figure 4B). We did not observe a substantial deviation in the number of stroke patients treated with thrombectomy (average of 24 per month in 2019, 29 in March 2020, and 19 in April 2020).

## Discussion

In three different datasets from the metropolitan area of Berlin and the rural region of Northeastern Germany, the weekly number of stroke and TIA patients presenting to hospitals declined sharply in the early phase of the COVID-19 pandemic. Patients admitted during this period had more severe strokes compared with preceding years. While the weekly mean average of patients who received thrombolysis declined during the early pandemic, the proportion of patients treated with thrombolysis and numbers of performed thrombectomies were similar to the historical averages. The decline in stroke admissions occurred parallel to media reports of increasing numbers of Sars-CoV-2 infections in other European countries and reached its maximum shortly after nationwide decrees restricting physical contacts were issued. Notably, this decline occurred before a relevant increase of Sars-CoV-2 infections and COVID-19 deaths was seen in Berlin and Northeastern Germany. Admission numbers in the emergency department of the Charité in Berlin increased again to near-normal levels after a low plateau of 4 weeks while restrictions were still in place.

In our primary analysis, we assessed admissions of stroke and TIA patients to 18 hospitals in an urban and a rural region in Northeastern Germany. Our data sources are derived from local registries and included only confirmed stroke and TIA diagnoses with a high degree of data completeness. In addition, our emergency department data sources providing information on age, stroke severity, and thrombolysis allowed us to characterize the population of affected stroke patients in more detail in the secondary analyses.

Declines in hospital admissions for stroke and TIA during the COVID-19 pandemic have also been reported from China (4) (≈40% fewer admissions) and Spain (5, 6) (≈23–25% fewer admissions). Our results add new information and indicate that patients with no or only mild deficits were less often admitted during the early local COVID-19 pandemic period, suggesting that these patients tended to avoid hospitalization. It is tempting to speculate that fear of infection with Sars-CoV-2 was the major reason. In contrast to patients with TIA and minor stroke, patients with moderate or severe stroke may feel a much higher need to seek appropriate medical care and therefore may have still presented to hospitals despite fear of infection with Sars-CoV-2. Decreased social support hampering the detection of stroke may also have contributed to the decline in stroke cases. Our findings do not indicate a relevant deviation of hospital attendance according to age compared with previous years' weekly averages. Also, in contrast to studies from China (4) and France (7), and suggested by data on decreasing use of an imaging software to support thrombectomy decisions (8), we did not observe a relevant deviation of numbers of patients receiving thrombectomy compared with historical averages. While the number of patients treated with thrombolysis declined during the early pandemic, the proportion of patients with thrombolysis did not show a relevant deviation compared to historical averages in our study, which is probably attributable to the overall lower number of admissions in the same period.

The metropolitan area of Berlin and the region of Northeastern Germany were affected by relatively low numbers of Sars-CoV-2 infections compared to other European regions (in particular Northern Italy, Madrid and Catalonia in Spain, Paris and Grand-Est in France) and the United States (State of New York). The comparatively low numbers of local Sars-CoV-2 infections in Northeastern Germany (total of 12,115 confirmed infections as of June 23, 2020 in the three German states of Berlin, Brandenburg, and Mecklenburg-Western Pomerania with 6.8 million inhabitants) have not overwhelmed the regional health systems so far. In Northeastern Germany, the need for ICU beds has not exceeded the ICU capacity; measures to rapidly increase the capacity were executed in the early pandemic phase. Even during the time of highest COVID-19 related ICU occupancy at the Charité on April 26, 2020, there were 24% fewer hospitalized patients in the Charité compared to the year before (internal statistics). Although the German government ordered hospitals to suspend all elective hospital admissions on March 18 in order to reserve hospital beds for expected COVID-19 patients, TIA and stroke patients have always been regarded as medical emergencies in emergency departments with no policy for outpatient workup. Therefore, while public health measures were successful in containing the spread of Sars-CoV-2 infections, they may have led to collateral damage on medical care for a subgroup of stroke patients with TIA and minor stroke who may have stayed at home rather than to seek medical help.

Interestingly, our emergency department data indicate that this decline in admissions may be transient as the number of stroke patients returned to near normal levels after 4 weeks although restrictions were still in place and the numbers of hospitalized COVID-19 patients increased. Our descriptive analyses suggest the need for public communication strategies to ensure that patients with stroke or TIA (and supposedly other critical disease) symptoms present to hospitals and receive adequate diagnostic evaluation and treatment despite an ongoing health emergency. After noticing a drop in stroke and heart attack cases, the Charité hospital informed the public on March 29, 2020, that patients with stroke or heart attack symptoms should immediately contact the emergency services (9). This may have contributed to the observed increase of stroke admissions afterwards. Measures need to be taken to ensure safe transfer and evaluation of stroke patients and to inform the public about the risk of missed and untreated TIA and stroke.

There are several limitations of our study. First, we did not include all hospitals in Berlin and in Northeastern Germany; nevertheless, our data covers 9 of 15 stroke units in Berlin and a large area in rural Northeastern Germany, indicating that our data is representative for the regional situation of stroke admissions. Second, because of different regional spreads of Sars-CoV-2 and differences in health system capacities, our results may not be generalizable to other settings. Third, registry data regarding hospital admissions used in the primary analyses was only available until to the end of March 2020. This lag is caused due to the nature of these lists as they are based on hospital main diagnosis code at discharge. The data used in the secondary analyses from the emergency department stroke logs are updated in real time, and therefore, were available through May 12, 2020, affording us more detailed insights as the pandemic situation unfolded. Specifically, after the initial decline in the early phase, we observed increasing numbers of stroke cases in the second half of April. Further research should investigate the evolution of stroke numbers beyond the early phase of the COVID-19 pandemic. It is unclear whether fewer hospital admissions for TIA and minor stroke will result in a higher frequency of more severe strokes in the weeks or months afterwards attributable to delayed secondary prevention. Finally, our study does not provide data on reasons why patients may have avoided hospitals.

In summary, we observed a relevant deviation of the weekly numbers of stroke and TIA patients presenting to hospitals in the early local phase of the COVID-19 pandemic in Berlin and Northeastern Germany compared with historical averages. Our results may inform health officials and authorities that containment measures should be paralleled by measures to ensure adequate awareness, diagnostic workup and treatment for serious diseases that may present with minor symptoms, such as stroke, which can have major consequences if not addressed early.

## Data Availability Statement

The raw data supporting the conclusions of this article will be made available by the authors, without undue reservation.

## Ethics Statement

Ethical review and approval was not required for the study on human participants in accordance with the local legislation and institutional requirements. Written informed consent for participation was not required for this study in accordance with the national legislation and the institutional requirements.

## Author Contributions

HE, BS, CL, CF, JR, JS, CN, ME, and HA: conception and design of the work. All authors: acquisition, analysis, or interpretation of data for the work, drafting the work or revising it critically for important intellectual content, and final approval of the version to be published.

## Conflict of Interest

JR's position is funded by a grant from the Else-Kröner-Fresenius Foundation (www.ekfs.de, GSO/EKFS-17). She also reports financial support from Novartis Pharma for a self-initiated research project on migraine remission in aging on a population level unrelated to this work. CN reports lecture fees paid from Boehringer Ingelheim, Bristol-Myers Squibb, Pfizer Pharma, Abbott and Gore. ME received funding from DFG under Germany's Excellence Strategy – EXC-2049 – 390688087, BMBF, DZNE, DZHK, EU, Corona Foundation, and Fondation Leducq. He also reports grants from Bayer and fees paid to the Charité from Bayer, Boehringer Ingelheim, BMS, Daiichi Sankyo, Amgen, GSK, Sanofi, Covidien, Novartis, Pfizer, all outside the submitted work. HA reports being the principal investigator of the ANNOTeM network, funded by the German Innovation Funds and having acquired funding for the B-SPATIAL Registry by the German Research Foundation (DFG). He also reports having received speaker and consultancy honoraria by Bayer Healthcare, BMS, Boehringer Ingelheim, Novo-Nordisk, Pfizer and Takeda. The remaining authors declare that the research was conducted in the absence of any commercial or financial relationships that could be construed as a potential conflict of interest.
