# Supplementary material for: Stroke Admissions, Stroke Severity, and Treatment Rates in Urban and Rural Areas During the COVID-19 Pandemic
Source: Front Neurol. 2021 Jan 6;11:607193. doi: 10.3389/fneur.2020.607193 (PMC7815522; doi:10.3389/fneur.2020.607193)
Supplement: Supplementary file 1 [file Data_Sheet_1.DOCX]

Supplemental Material

## Supplemental Methods

### Maps of participating regions

### *Supplemental Figure I:* B-SPATIAL registry from the Berlin metropolitan region
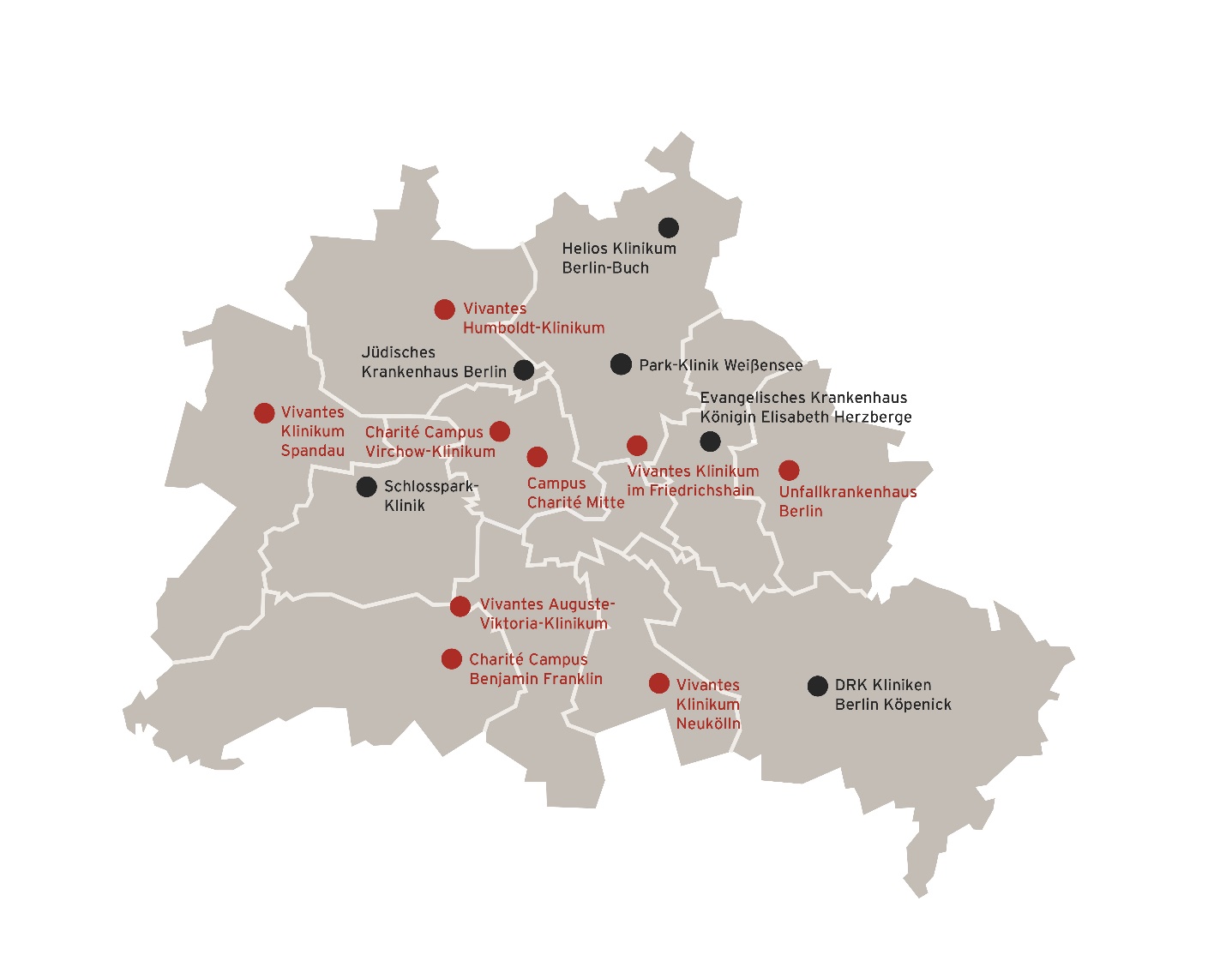


Supplemental Figure I. B-SPATIAL: Berlin - SPecific Acute Treatment in Ischemic or hAemorrhagic Stroke With Long Term Follow-up) registry. Data for the current study was available from the Charité hospitals, Vivantes hospitals, and Unfallkrankenhaus Berlin (red). The DRK-Kliniken Berlin Köpenick, Evangelisches Krankenhaus Königin Elisabeth Herzberge, Helios Klinikum Berlin-Buch, Jüdisches Krankenhaus Berlin, Park-Klinik Weissensee, Schlosspark-Klinik (black) are also part of the B-SPATIAL registry but data was not available from these hospitals for the current study.

*Supplemental Figure II:* ANNOTeM TeleNeurology network in rural areas in Northeastern Germany

####
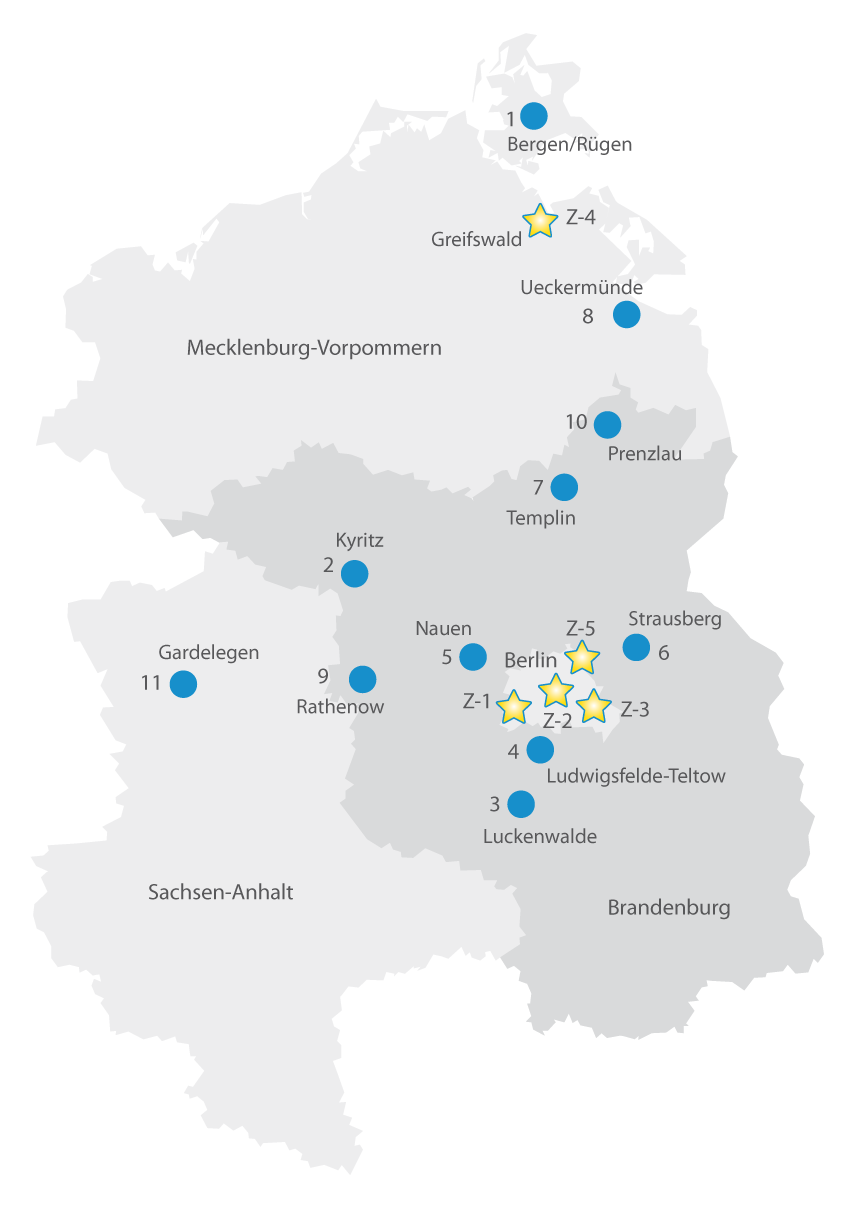


Supplemental Figure II. ANNOTeM: AkutNeurologische Versorgung in NOrdost-Deutschland mit TEleMedizinischer Unterstützung. Data from (1) Sana-Krankenhaus Bergen/Rügen (Mecklenburg-Western Pomerania), (3) KMG-Klinikum Luckenwalde (Brandenburg), (4) Evangelisches Krankenhaus Ludwigsfelde-Teltow (Brandenburg), (5) Havelland-Kliniken Nauen (Brandenburg), (6) Krankenhaus Märkisch-Oderland Strausberg (Brandenburg), (7) Sana-Krankenhaus Templin (Brandenburg), (8) AMEOS-Klinik Ueckermünde (Mecklenburg-Western Pomerania), (9) Havelland-Kliniken Rathenow (Brandenburg), (10) Kreiskrankenhaus Prenzlau (Brandenburg) were included in the current study. The (2) KMG-Klinikum Kyritz (Brandenburg) and the (11) Altmark-Klinikum Krankenhaus Gardelegen (Saxony-Anhalt) are also participating hospitals in the TeleNeurology Network but data was not available from these hospitals for the current study. Z1-Z5 depict the hubs of the network (Z1+Z2: Charité – Universitätsmedizin Berlin, Z-3: Unfallkrankenhaus Berlin, Z-4: Universitätsmedizin Greifswald, and Z-5: Epilepsieklinik Tabor Bernau).

## Supplemental Results

These supplemental results display the same graphs as the main paper, but instead of applying a rolling average of 3 weeks, we display the values for single weeks with confidence intervals. The 95% confidence intervals (CI) for the number of stroke patients are based on rate ratios, assuming that the catchment area and the corresponding source population of the corresponding hospitals was stable over time. The 95% confidence intervals for percentages are based on risk ratios, while the 95%CI for the ratio of the two NIHSS values is based on approximated standard errors for unpaired data.

*Supplemental Figure III:*

Supplemental Figure III. Stroke and TIA admission numbers of (upper panel) nine stroke units in the metropolitan area of Berlin and (lower panel) nine hospitals of a TeleNeurology Network in Northeastern Germany 2018–2020. Admission numbers are plotted against week numbers. All individual years are plotted in grey. The weekly average of 2018–2019 is set at 100%. The year 2020 is plotted in red with weekly corresponding 95% confidence intervals in pink. Blue inlays depict cumulative confirmed Sars-CoV-2 infections and COVID-19 deaths in the respective weeks.

*Supplemental Figure IV:*

Supplemental Figure IV. Stroke and TIA related emergency department visits at the University hospital Charité in Berlin 2016–2020. The weekly average of 2016–2019 is set at 100%. The year 2020 is plotted in red with weekly corresponding 95% confidence intervals in pink. Blue inlay depicts cumulative confirmed Sars-CoV-2 infections and COVID-19 deaths in the respective weeks.

Supplemental Figure V:

Supplemental Figure V. (Upper panel) NIHSS on admission and (lower panel) proportion of patients with moderate to severe stroke (NIHSS>5) at the University hospital Charité in Berlin 2016–2020. The weekly average of 2016–2019 is set at 100%. The year 2020 is plotted in red with weekly corresponding 95% confidence intervals in pink. Blue inlays depict cumulative confirmed Sars-CoV-2 infections and COVID-19 deaths in the respective weeks.

Supplemental Figure VI:

Supplemental Figure VI. (Upper panel) Proportion of patients ≤65 years and (lower panel) proportion of patients treated with thrombolysis at the University hospital Charité in Berlin 2016–2020. The weekly average of 2016–2019 is set at 100%. The year 2020 is plotted in red with weekly corresponding 95% confidence intervals in pink. Blue inlays depict cumulative confirmed Sars-CoV-2 infections and COVID-19 deaths in the respective weeks.
